# Assessment of *Beauveria bassiana* as an endophyte of maize and its effects on plant growth

**DOI:** 10.1371/journal.pone.0342175

**Published:** 2026-02-12

**Authors:** Abigail L. Kropf, Aaron J. Gassmann

**Affiliations:** Department of Plant Pathology, Entomology and Microbiology, Iowa State University, Ames, Iowa, United States of America; Osmania University, INDIA

## Abstract

*Beauveria bassiana* (Balsamo) Vuillemin (Hypocreales: Cordycipitaceae) is an insect pathogenic fungus that is well established as a microbial biopesticide. It occurs naturally in agricultural soil and can grow endophytically (i.e., within plant tissue) in a number of important crops, including maize, *Zea mays*. However, patterns of colonization of maize tissues by *B. bassiana*, and effects on growth of maize plants, are not well characterized. We assessed interactions of two strains of *B. bassiana* with maize. One isolate, GHA, is a well-studied strain and the other, MA20, was brought into culture from an insect cadaver. Maize plants were exposed to *B. bassiana* by inoculating seeds with fungal conidia prior to planting. This study had three objectives. 1) To test how the presence of *B. bassiana* affects plant growth. 2) To assess the capacity of *B. bassiana* to grow endophytically in plant tissues, specifically root, stem and leaf tissue. 3) To measure the persistence of *B. bassiana* in the growth substrate. We did not detect any significant effects of *B. bassiana* on growth of maize. However, we did find that inoculating maize seeds with *B. bassiana* led to endophytic colonization of root, stem and leaf tissues, with the occurrence of *B. bassiana* colonizing root tissue significantly more than stem and leaf tissue, and stem tissue significantly more than leaf tissue. Additionally, we found evidence for the persistence of *B. bassiana* in the growth substrate of maize plants. These results provide a better understanding of the interaction of maize with *B. bassiana* and may aid in the development of approaches to manage pests of maize. In particular, endophytic colonization of plants by *B. bassiana* can alter interactions of plants with fungal pathogens and insect pests, with research in these areas offering a next step to build on the research described in this study.

## Introduction

Entomopathogenic fungi are a diverse group with the common attribute of causing disease in arthropods, including insects and mites, and those in the order Hypocreales are ubiquitous in the environment, with individual species often employing various life-history strategies [1]. For example, in addition to being insect pathogens, many can live saprophytically, facilitating persistence outside of an insect host; while in other instances they may form tightly knit relationships with plants [2]. *Beauveria bassiana* (Balsamo) Vuillemin (Hypocreales: Cordycipitaceae) is an entomopathogenic fungus widely studied for its applications in pest management because of its ability to kill insect pests [1]. Additionally, *B. bassiana* has the potential to form complex relationships with plants through its occurrence in the rhizosphere, which is defined as the interface where plants interact with the biological, physical, and chemical components of the soil [1,3,4]. *Beauveria bassiana* can also grow epiphytically (i.e., on the surface of tissue) or endophytically (i.e., within plant tissue) [5]. While the capacity of *B. bassiana* to occur as an endophyte has been previously reported, less is known about the process of endophytic colonization or the effects of this endophytic colonization on plant growth [5–7].

Entomopathogenic fungi can indirectly affect plant growth by shaping the soil environment and, more directly, by forming associations within the rhizosphere or within root tissue. Effects may arise from alteration of soil conditions through the secretion of enzymes that can degrade complex substrates such as chitin and lignin, and subsequentially may increase the availability of nutrients to plants [8]. Beyond altering nutrient availability, entomopathogenic fungi may affect plant growth by forming associations within roots. For example, the entomopathogenic fungus *Metarhizium robertsii* (Hypocreales: Clavicipitaceae) has been shown to behave similarly to arbuscular mycorrhiza in some studies, in which the fungal partner helps the plant access nutrients such as nitrogen in exchange for photosynthetic products [9].

Some fungal endophytes have been shown to increase plant growth through the upregulation of the photosynthetic capabilities of plants [10]. In some cases the occurrence of entomopathogenic fungi, including endophytic *B. bassiana*, has been shown to promote plant growth in crops, including maize, cotton, and sorghum, although the mechanisms are not well understood [6,11–16]. However, the extent to which *B. bassiana* might promote plant growth may not be universal, instead, it may vary based on the fungal strain, the species of host plant, and abiotic factors such as nutrient availability [17,18].

In addition to effects on plant growth, endophytic colonization of plants by entomopathogenic fungi can alter interactions of plants with both herbivorous insects and plant pathogens. Endophytic *B. bassiana* has been previously shown to deter or reduce feeding by insect pests and increase plant resistance to herbivory in various crops including maize, tomato and tobacco [6,14,19–21]. In some cases, fungal metabolites produced within plant tissue deter herbivores or reduce the development of insects that feed on plant tissue [1,22,23]. Additionally, the establishment of entomopathogenic fungi in the rhizosphere or as an endophyte may reduce injury to plants from plant pathogens through competitive exclusion, the production of secondary metabolites, or by inducing plant defenses [17,24,25]. Entomopathogenic fungi as endophytes have become an increasingly important area of study because they may provide dual protection of plants from insect pests and plant pathogens [1,25].

Maize is a crop of great economic importance in the United States, with an average of 36 million hectares planted to maize each year [26]. Several insect pests can substantially reduce maize yields, and past research suggests that entomopathogenic fungi, including *B. bassiana*, may aid in the management of some of these pests [6,27,28]. *Beauveria bassiana* has been documented in maize fields [29], and has been shown to kill maize pests such as larvae of western corn rootworm (*Diabrotica virgifera virgifera*) [30]. Furthermore, *B. bassiana* has been shown to colonize maize endophytically and promote growth of maize plants [6,7,16,28]. These data suggest that *B. bassiana*, as an endophyte, may increase plant growth while serving to reduce feeding injury from some insect pests.

Entomopathogenic fungi, including *B. bassiana*, are noteworthy because of their multifaceted ecological roles, with life-history strategies that include their presence in the environment as a saprophyte, endophyte, entomopathogen, and inhabitant of the rhizosphere. These interactions may be important for crop plants, affecting both their growth and their interactions with pest species. The goals of this study were to characterize interactions of maize with *B. bassiana*, specifically, the effects on the growth of maize plants, the extent to which *B. bassiana* can grow as an endophyte, and the capacity of *B. bassiana* to persist in the rhizosphere.

## Methods

In this study, maize seeds were exposed to one of two strains of *Beauveria bassiana*, GHA and MA20, and data were collected on plant growth, the presence of *B. bassiana* in the rhizosphere, and the occurrence of *B. bassiana* growing endophytically. Two concentrations of *B. bassiana* conidia were tested for each strain, and data were collected at each of the two time points.

### Fungal strains

*Beauveria bassiana* GHA was initially provided by the United States Department of Agriculture, Agricultural Research Service, Northern Plains Agricultural Research Laboratory (Sydney, Montana). A second strain of *B. bassiana*, MA20, was isolated from a mycosed western corn rootworm adult collected from a maize field in Story County, Iowa in 2020. Initial diagnosis of the fungus as *B. bassiana* was based on fungal morphology on the mycosed cadaver and morphology of fungal conidia [31]. To initiate culturing of MA20, conidia produced on the insect cadaver were grown on a selective medium described in Chase et al. [32]. This medium is selective for *B. bassiana* (i.e., permits the growth of *B. bassiana* while inhibiting the growth of other microorganisms)*,* and consists of 2.0% oatmeal agar with 0.62g/ L of dodine (Syllit 65W, Platte Chemical Inc., Greenville, MS), 0.25g/ L chloramphenicol (C0378, Sigma, Saint Louis, MO), and 10 mg/ L crystal violet (C6158, Sigma, Saint Louis, MO). Conidia produced on this selective media then were harvested and used to infect and kill larvae of the greater wax moth, *Galleria mellonella* (Lepidoptera: Pyralidae)*.* In summary, the diagnosis of MA20 as a strain of *B. bassiana* was based on 1) morphology of the fungus on mycosed cadavers, 2) morphology of fungal conidia, 3) capacity to grow on the selective media of Chase et al. [32], and 4) capacity to kill larvae of *G. mellonella*.

### Production of conidia

Conidia used in this project from GHA and MA20 were produced on potato dextrose agar (Becton, Dickinson and Company, Sparks, MD) with 0.15% chloramphenicol (C0378, Sigma, Saint Louis, MO), which was held in a Petri dish. Once inoculated, plates were stored at 28^o^C for 14–17 days, with fungal conidia checked for viability 24 hours before use in experiments, following techniques from Goettel and Inglis [33]. Each strain was propagated by infecting *G. mellonella* after every three to four rounds of culturing on potato dextrose agar, and this was done to ensure that the fungal strains maintained their pathogenicity to insects. Conidia used in these experiments were produced on PDA without passing through an insect in order to minimize variation of isolates among experimental replicates.

### Experimental approach

Maize seeds (Viking U42-92, Albert Lea, MN), which lacked any type of pesticidal seed treatment, were surface sterilized following Clifton et al. [34]. In brief, maize seed was agitated in a solution 0.1% Tween 80 (Tween 80, Acros Organics, Morris Plains, NJ) for 30 s; followed by being placed in a solution of bleach (AROCEP Ultra Bleach, 6% hypochlorite, Woodridge, IL), diluted to 2% hypochlorite, for 2 min; and finally seeds were soaked in a solution of 70% ethanol for 30 s; after which, seeds were rinsed twice in deionized water. Seeds were allowed to dry for two hours before being placed individually in 5 ml microcentrifuge tubes (MacroTube 5, MTC Bio, Sayreville, NJ) and soaked in a suspension of fungal conidia or in a control solution that lacked conidia. Sterilized seeds were not assessed for the presence of microbes prior to exposure to fungal conidia. Each seed was soaked for 24 hours, individually, in 1 ml of a solution which contained 0.5 of 15% agar (Fisher Scientific Pittsburg, PA) and 0.5 ml of Tween 80 that contained either 1) 3 × 10^8^ conidia of *B. bassiana* per seed, 2) 3 × 10^9^ conidia of *B. bassiana* per seed or 3) an experimental control that did not contain any fungal conidia. The resulting solution produced a gel consistency that allowed the conidia to remain in suspension during the 24 h period.

Maize plants were grown in 164 ml cylindrical containers (height = 20.95 cm; diameter = 3.8 cm) (SC10 – Ray Leach “Super Cell” Air Pruning, Stuewe & Sons Inc, Tangent, Oregon), with one container nested within a second container and a piece of mesh cloth (Poly Chiffon, Hobby Lobby Stores Inc., Oklahoma City, OK) placed between containers to prevent the loss of the growth substrate while allowing excess water to drain from the containers. The growth substrate, vermiculite (VWR International, Radnor, PA), was sterilized in an autoclave at 121^o^C and 22 psi for 1 hour and allowed to cool. Sterilized vermiculite was added until it filled 75% of the container, then lightly compressed and additional vermiculite added to fill the container to the top, with vermiculite lightly compressed again until it was within ca. 3 cm from the top of the container. Vermiculite was thoroughly moistened (i.e., water was added until it dripped from the bottom of the containers) before maize seeds were planted. A cylindrical opening, approximately 3 cm deep, was made for each seed in the center of the container, and a single seed was placed in the center of the opening, with the vermiculite then replaced to cover the seed before being lightly compressed.

Maize seeds were planted immediately after they were removed from the tween-agar solution. Plants were grown in a greenhouse (16:8 h light/dark; 28°C) with supplemental lighting provided by high-pressure sodium bulbs (PL3000, P.L Light Systems, Hamilton, ON, Canada). Once seeds germinated, they were watered as needed. Seven days after planting, each plant received 20 ml of a fertilizer solution (N:P:K, 24:8:16) (Miracle Gro Soluble All-Purpose Plant Food, 2.8 g/L of water). Fourteen days after planting, plants not destructively sampled received an additional 20 ml of fertilizer.

For this study, GHA and MA20 were assessed in two separate experiments. Each experiment consisted of four blocks conducted over time. For each block, seeds were treated with one of three concentrations of conidia: 3 × 10^8^, and 3 × 10^9^ conidia/seed, and a control of 0 conidia/seed, with 12 plants grown per concentration of conidia in each block, for a total of 36 plants per block and 144 plants per fungal strain (36 plants per block × 4 blocks). However, across the eight blocks of the study, 13 seeds did not germinate, eight plants from blocks with GHA and five plants from blocks with MA20, which reduced the total sample size to 136 plants for the experiment with GHA and 139 plants for the experiment with MA20.

Fourteen days after planting, half of the plants from each treatment were randomly selected and measured for plant height and basal diameter prior to being destructively sampled to collect data on either plant biomass or to assess the incidence of *B. bassiana* colonization. Twenty-one days after planting, all remaining plants were sampled, with half of the plants selected at random to assess the incidence of *B. bassiana* colonization and the remaining plants used for measurements of plant biomass.

### Measurements of plant size

Measurements were taken on plant height and basal diameter for all plants. Height was measured from the point where the stem emerged from the vermiculite to the tip of the longest leaf when fully extended. The basal diameter was measured at the point where the stem emerged from the vermiculite.

For plants used to assess biomass, aboveground plant tissue was removed, placed in a small envelope and held in a drying oven (Thermo Fisher Scientific, Marietta, OH) at 60°C for 2 wks. After removing the aboveground biomass, the root mass was removed from containers and placed on top of a stack of two sieves of different sizes (No.5 sieve; opening = 4.0 mm on top of a No. 30 sieve; opening = 0.6 mm; Hogentogler & Co., Inc., Columbia, MD) with the vermiculite washed away using a stream of water, and all root tissue collected. Large roots were collected from the top sieve (No. 5), while fine roots that passed through the top sieve were collected in the bottom sieve (No. 30). After removing any vermiculite, root tissue from an individual plant was placed into an envelope and held in a drying oven at 60°C. After 2 wks, aboveground biomass and root tissue were removed from the drying oven and set on a laboratory bench for at least 1 wk, to equilibrate with ambient laboratory conditions, before measuring dry mass to the nearest 0.1 mg on an analytical balance (XS205 Dual Range Balance, Mettler Toledo, Greifensee, Switzerland). A total of 64 plants were used to assess dry mass for GHA and 67 plants were used to assess dry mass for MA20.

### Assessment of endophytism

Once measurements on plant height and basal diameter were taken, plants not used to assess aboveground and belowground biomass were used to test whether *B. bassiana* grew endophytically (i.e., grew within plant tissue) in the roots, stem, and leaves of maize plants. The basic approach followed Clifton et al. [34] and consisted of excising a portion of the plant tissue, sterilizing the surface of the tissue, and then placing it on the growth medium of Chase et al. [32], as described under Fungal strains, which permits the growth *B. bassiana* but inhibits the growth of other microorganisms (i.e., is selective for *B. bassiana*). The plant tissue sampled for endophytic *B. bassiana* consisted of the largest fully formed leaf, stem tissue 1 cm above the vermiculite, and the radical root. One sample of each type of plant tissue was taken from each plant used in this assessment.

Starting with control plants, which were not exposed to *B. bassiana*, plant tissue was surface sterilized through a series of washes following Clifton et al. [34]. In brief, plant tissues were moved individually through a series of beakers for the following amounts of time 1) 30 s in sterile 0.1% Tween 80, 2) 2 min in a 2% hypochlorite solution, 3) 30 s in 70% ethanol, and 4) two consecutive rinses, of 30 s each, in sterile deionized water. Tissue was then checked to confirm that the surface had been sterilized by pressing and then removing the plant tissue from an agar solid (39% potato dextrose agar), held in a Petri dish, with the agar then observed for growth *of B. bassiana* over a period of 14 days. Each piece of maize tissue was checked in this manner, with one Petri dish used for each sample of plant tissue. Both sides of a maize leaf were pressed into agar, while the root and the stem were rolled across the agar. The presence of *B. bassiana* on the potato dextrose agar would indicate that the sample was not successfully surface sterilized and that any resulting data on endophytism should be excluded from analysis.

**Fig 1:**
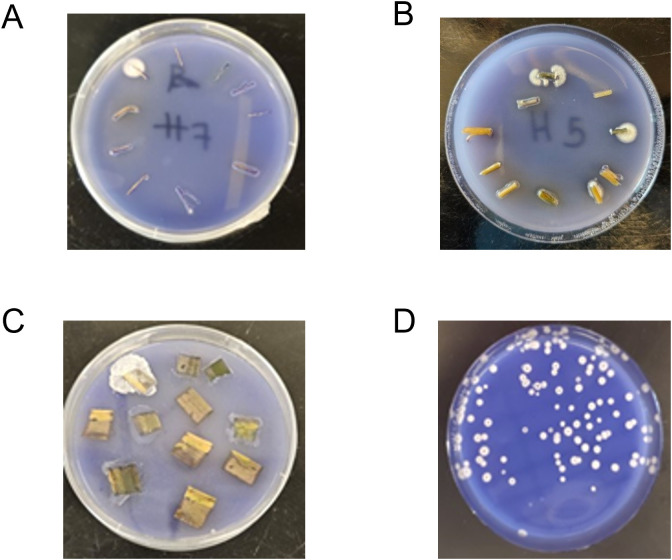
*Beauveria bassiana* recovered from maize tissue and vermiculite. Photographs capturing growth of endophytic *Beauveria bassiana* from three types of corn tissue: A) roots B) stems C) leaves; and a photograph D) of colony forming units of *B. bassiana* which grew from a sample of vermiculite.

After plant tissue was surface sterilized, each sample of plant tissue was aseptically cut into ten pieces, with each set of ten pieces then placed in a single Petri dish (diameter = 100 cm) containing the previously described *Beauveria*-selective medium of Chase et al. [32]. Leaf tissue was cut into ten 1 cm × 1 cm squares and placed on selective media in a single Petri dish. Stem tissue was cut in half, lengthwise, and then cut into ten pieces 1 cm long and placed, pith side down, on selective media. Because the roots were very thin, they were cut into 10 pieces, 1 cm long, and scored down the center to expose the inside of the root, after which root pieces were placed on selective media with the incision facing downward. Plates containing plant tissues were sealed with parafilm (PM-996, Bemis, Neenah, WI) and stored in a biological incubator (Percival, Perry IA) (27^o^C, 0:24 h L:D). Each plate was scored for the presence or absence of endophytic *B. bassiana* after two weeks of incubation. If *B. bassiana* growth was present on the selective media, extending from any of the ten pieces of tissues, it was scored as present for that tissue from that plant (Figs 1A to 1C). Fungal outgrowth from plant tissue was confirmed as *B. bassiana* through 1) its ability to grow on a selective media and 2) by observing morphology consistent with *B. bassiana.* A total of 72 plants were assessed for the presence of endophytism in each experiment.

### Assessment of *B. bassiana* in the rhizosphere

For plants used to test for the presence of *B. bassiana* growing endophytically, we also sampled vermiculite to test for the presence of *B. bassiana* in the rhizosphere. For each plant, vermiculite was removed from the roots and then thoroughly mixed, after which a single 1 ml sample of vermiculite was collected. This sample was placed in a 15 ml centrifuge tube and mixed with 9 ml of a 0.1% Tween 80 solution, before being diluted in a 1:10 ratio in 0.1% Tween 80 held in a 15 ml centrifuge tube (Nunc, Thermo Fisher Scientific, Marietta, OH). The diluted suspension was then thoroughly vortexed, and a single sample of 20 µl was spread on selective media of Chase et al. [28] held in a Petri dish (size = 100 cm). One Petri dish was assessed per plant, with a total of 72 Petri dishes assessed for colony-forming units per experiment. Two weeks thereafter, the number of colony-forming units on the selective media in each Petri dish were counted. Colony-forming units provide a measure of the number of viable *B. bassiana* propagules (e.g., conidia) in a sample (Fig 1D).

### Data analysis

Data were analyzed using SAS 9.4 (SAS Institute Inc., Cary, NC), with data for GHA and MA20 analyzed separately. Root-to-shoot ratio was calculated as the quotient of root biomass divided by aboveground biomass. Plant size metrics of plant height, basal diameter, aboveground biomass, root biomass, and root-to-shoot ratio were analyzed with a mixed-model analysis of variance (ANOVA) (PROC MIXED). Fixed factors in the analysis included treatment (0, 3 × 10^8^, and 3 × 10^9^ conidia/seed), time (14 days after planting and 21 days after planting), and the interaction of treatment and time. Random factors included block, block × treatment, block × time, and block × treatment × time. Plant height, basal diameter, aboveground biomass, and root biomass were transformed with a square-root function, and root-to-shoot ratio was log_10_ transformed before analysis to normalize residuals.

Binary data on the presence versus absence of *B. bassiana* as an endophyte were analyzed with a test of independence using logistic regression (PROC CATMOD). The occurrence of *B. bassiana* was analyzed separately by type of plant tissue (i.e., roots, stems and leaves) in an analysis that included the factors of treatment (3 × 10^8^ and 3 × 10^9^ conidia/seed), time (14 d after planting and 21 d after planting) and their interaction. Each type of plant tissue was analyzed separately to guard against inflating the degrees of freedom in the statistical model, which could occur if multiple plant tissues from the same plant were analyzed in a single model. This same statistical model was applied to analyze the presence of endophytic *B. bassiana* in each plant (i.e., its presence in any plant tissue).

The occurrence of endophytic *B. bassiana*, pooled by treatment and time, was compared between tissue types (i.e., roots *vs* stems *vs* leaves) using a χ^2^ test of independence (PROC FREQ). Additionally, we compared plants that received a treatment of *B. bassiana* conidia (3 × 10^8^ and 3 × 10^9^ conidia/seed) to the control, at both 14 and 21 d, based on a one-tailed test of independence with a Fisher’s exact test (PROC FREQ). The null hypothesis was that there was no difference in the frequency of plants with endophytic *B. bassiana* between a treatment (i.e., 3 × 10^8^ or 3 × 10^9^ conidia/seed) at a given time point (i.e., 14 or 21 d post planting) and the control (seeds that were not treated with *B. bassiana* conidia). The alternative hypothesis was that the frequency of plants colonized by *B. bassiana* was greater for a treatment than the control.

The number of colony-forming units per Petri dish was multiplied by 50 to calculate the number of colony-forming units per 1 ml of the diluted sample and then multiplied by the dilution factor (i.e., 100) to calculate the number of colony-forming units per ml of vermiculite. Data on the number of colony-forming units per ml of vermiculite were log_10_ transformed and analyzed with a mixed-model analysis of variance (ANOVA) (PROC MIXED). Fixed factors in the analysis included treatment (3 × 10^8^ and 3 × 10^9^ conidia/seed), time (14 d after planting and 21 d after planting), and the interaction of treatment and time. Random factors included block, block × treatment, block × time, and block × treatment × time. Since *B. bassiana* was not recovered from the vermiculite of control plants, a nonparametric Kruskal Wallis test (PROC NPAR1WAY) was used to compare plants treated with conidia (3 × 10^8^ and 3 × 10^9^ conidia/seed) to the control based on a one-tailed test. The null hypothesis was that there was no difference in the colony forming units of *B. bassiana* for each combination of treatment (3 × 10^8^ or 3 × 10^9^ conidia/seed) at each time point (14 or 21 d after planting) versus the control (seeds that were not treated with *B. bassiana* conidia). The alternative hypothesis was that the number of colony-forming units was greater for a treatment group than the control.

## Results

Metrics for plant size increased over time but did not differ between the *B. bassiana* treatments and the no-inoculum control. In the case of the experiment with *B. bassiana* GHA, plant height, basal diameter, aboveground biomass and root biomass all increased significantly over time (Tables 1 and 2). The same pattern was observed for the experiment with *B. bassiana* MA20 (Tables 3 and 4). There was also a significant effect of time on root-to-shoot ratio (i.e., quotient of root biomass divided by aboveground biomass) for both GHA (F = 1.38; df = 2,6; P = 0.321) and MA20 (F = 0.394; df = 2,6; P = 0.081) (Tables 1 and 3). For both experiments, root-to-shoot ratio decreased significantly over time (Tables 2 and 4). However, we did not detect a significant effect of treating seeds with conidia of *B. bassiana* compared to the no-inoculum control or a significant interaction of treatment with time for any of the plant metrics, and this was the case for both GHA and MA20 (Tables 1 and 3).

**Table 1:** Analysis of variance for effect of *Beauveria bassiana* GHA and time on growth metrics for maize plants.

Measurement	Effect	df	F-Statistic	P
**Root:shoot** ^**1**^	Treatment^2^	2, 6	1.38	0.321
	Time^3^	1, 3	93.81	0.002
	Treatment × Time	2, 6	0.76	0.509
**Aboveground Biomass**	Treatment	2, 6	1.49	0.299
	Time	1, 3	36.86	0.009
	Treatment × Time	2, 6	0.59	0.582
**Root Biomass**	Treatment	2, 6	0.11	0.894
	Time	1, 3	16.61	0.027
	Treatment × Time	2, 6	0.94	0.443
**Plant Height**	Treatment	2, 6	0.60	0.578
	Time	1, 3	44.86	0.007
	Treatment × Time	2, 6	1.74	0.254
**Basal Diameter**	Treatment	2, 6	0.83	0.479
	Time	1, 3	535.43	0.0002
	Treatment × Time	2, 6	0.75	0.512

^1^Ratio of root biomass to aboveground biomass.

^2^Treatment represents concentrations of *Beauveria bassiana* GHA tested: control of no conidia, 3 × 10^8^ conidia/seed, and 3 × 10^9^ conidia/ seed.

^3^Time represents the two time points at which data were collected: 14 days after planting and 21 days after planting.

**Table 2:** Various growth metrics for maize plants across time points and treatments with *Beauveria bassiana* GHA.

Measurement	Days after Planting^2^	Treatment^3^	Mean	S.E.
**Root:shoot** ^**1**^	14	Control	1.82	0.177
		3 × 10^8^	2.14	0.338
		3 × 10^9^	2.14	0.293
	21	Control	0.961	0.0792
		3 × 10^8^	1.19	0.139
		3 × 10^9^	1.01	0.0542
**Aboveground Biomass (g)**	14	Control	0.249	0.0291
		3 × 10^8^	0.227	0.0348
		3 × 10^9^	0.235	0.0355
	21	Control	0.777	0.0967
		3 × 10^8^	0.786	0.106
		3 × 10^9^	0.811	0.109
**Root Biomass (g)**	14	Control	0.434	0.0472
		3 × 10^8^	0.396	0.0462
		3 × 10^9^	0.438	0.0558
	21	Control	0.739	0.101
		3 × 10^8^	0.852	0.130
		3 × 10^9^	0.798	0.112
**Plant Height (cm)**	14	Control	29.7	1.45
		3 × 10^8^	26.6	1.72
		3 × 10^9^	26.2	2.03
	21	Control	38.6	2.00
		3 × 10^8^	40.1	1.75
		3 × 10^9^	40.3	1.77
**Basal Diameter (mm)**	14	Control	4.89	0.235
		3 × 10^8^	4.44	0.290
		3 × 10^9^	4.51	0.268
	21	Control	7.36	0.349
		3 × 10^8^	7.52	0.291
		3 × 10^9^	7.44	0.362

^1^Ratio of root biomass to aboveground biomass.

^2^Days after planting represents the two time points at which data were collected: 14 days after planting and 21 days after planting.

^3^Treatment represents concentrations of *Beauveria bassiana* GHA tested: control of no conidia, 3 × 10^8^ conidia/seed, and 3 × 10^9^ conidia/ seed.

**Table 3:** Analysis of variance for effect *of Beauveria bassiana* MA20 and time on growth metrics for maize plants.

Measurement	Effect	df	F-Statistic	P
**Root:shoot** ^**1**^	Treatment^2^	2, 6	3.94	0.081
	Time^3^	1, 3	93	<.0001
	Treatment × Time	2, 6	0.04	0.959
**Aboveground Biomass**	Treatment	2, 6	0.48	0.642
	Time	1, 3	42.21	0.007
	Treatment × Time	2, 6	1.98	0.218
**Root Biomass**	Treatment	2, 6	4.5	0.064
	Time	1, 3	19.99	0.021
	Treatment × Time	2, 6	3.49	0.099
**Plant Height**	Treatment	2, 6	0.25	0.789
	Time	1, 3	40.30	0.008
	Treatment × Time	2, 6	0.57	0.594
**Basal Diameter**	Treatment	2, 6	0.44	0.664
	Time	1, 3	124.11	0.002
	Treatment × Time	2, 6	1.72	0.257

^1^Ratio of root biomass to aboveground biomass.

^2^Treatment represents concentrations of *Beauveria bassiana* MA20 tested: control of no conidia, 3 × 10^8^ conidia/seed, and 3 × 10^9^ conidia/ seed.

^3^Time represents the two time points at which data were collected: 14 days after planting and 21 days after planting.

**Table 4:** Various growth metrics for maize plants across time points and treatments with *Beauveria bassiana* MA20.

Measurement	Days after Planting^2^	Treatment^3^	Mean	S.E.
**Root:shoot** ^**1**^	14	Control	2.12	0.151
		3 × 10^8^	2.35	0.146
		3 × 10^9^	2.59	0.364
	21	Control	1.27	0.103
		3 × 10^8^	1.40	0.0738
		3 × 10^9^	1.54	0.0827
**Aboveground Biomass (g)**	14	Control	0.132	0.0172
		3 × 10^8^	0.125	0.0098
		3 × 10^9^	0.115	0.0083
	21	Control	0.421	0.0585
		3 × 10^8^	0.457	0.0521
		3 × 10^9^	0.512	0.0512
**Root Biomass (g)**	14	Control	0.283	0.0435
		3 × 10^8^	0.291	0.0253
		3 × 10^9^	0.281	0.0194
	21	Control	0.521	0.0762
		3 × 10^8^	0.650	0.0917
		3 × 10^9^	0.810	0.110
**Plant Height (cm)**	14	Control	21.8	0.914
		3 × 10^8^	22.2	0.478
		3 × 10^9^	20.6	0.569
	21	Control	30.7	1.24
		3 × 10^8^	31.6	1.52
		3 × 10^9^	31.4	1.15
**Basal Diameter (mm)**	14	Control	3.55	0.198
		3 × 10^8^	3.61	0.126
		3 × 10^9^	3.47	0.096
	21	Control	6.76	0.291
		3 × 10^8^	6.72	0.251
		3 × 10^9^	7.29	0.171

^1^Ratio of root biomass to aboveground biomass.

^2^Days after planting represents the two time points at which data were collected: 14 days after planting and 21 days after planting.

^3^Treatment represents concentrations of *Beauveria bassiana* MA20 tested: control of no conidia, 3 × 10^8^ conidia/seed, and 3 × 10^9^ conidia/ seed.

**Fig 2:**
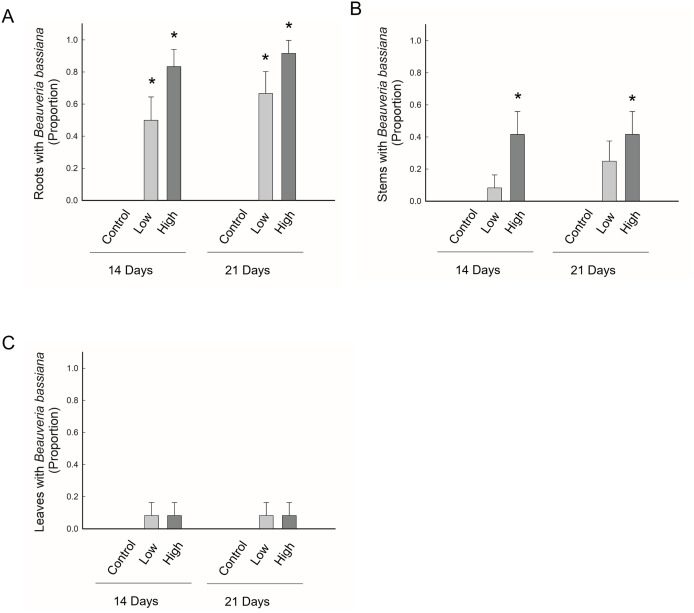
Endophytic occurrence of *Beauveria bassiana* GHA inA) roots, B) stems, and C) leaves. Data are presented for two time points, 14 and 21 days after planting, and for three concentrations of *B. bassiana* conidia that were used to inoculate maize seeds: control = 0 conidia/ seed, low = 3 × 10^8^ conidia/ seed, and high = 3 × 10^9^ conidia/ seed. Bar heights are the proportion of samples displaying endophytism, error bars are the standard error of the proportion. An asterisk indicates a significant difference from the control.

**Fig 3:**
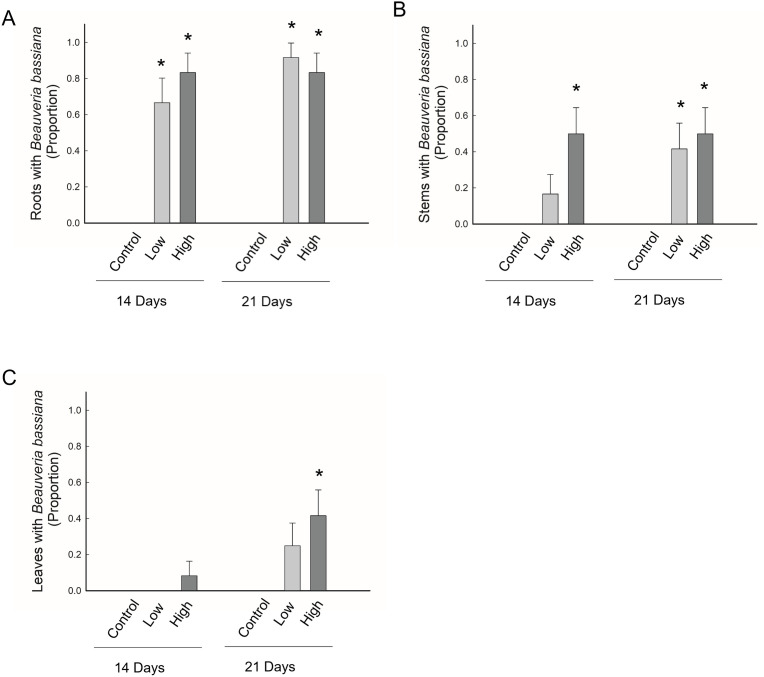
Endophytic occurrence of *Beauveria bassiana* MA20 in A) roots, B) stems, and C) leaves. Data are presented for two time points, 14 and 21 days after planting, and for three concentrations of *B. bassiana* conidia that were used to inoculate maize seeds: control = 0 conidia/seed, low = 3 × 10^8^ conidia/seed and high = 3 × 10^9^ conidia/ seed. Bar heights are the proportion of samples displaying endophytism, error bars are the standard error of the proportion. An asterisk indicates a significant difference from the control.

Petri dishes with potato dextrose agar, used to check the surface sterilization processes, were free of *B. bassiana*, indicating that any *B. bassiana* growing from plant tissue resulted from endophytic colonization by this fungus. Additionally, for control plants, which were not treated with *B. bassiana*, there was no evidence of endophytism by *B. bassiana* in any plant tissue (i.e., root, stem, or leaves) at either time point, and this was the case for experiments with both GHA and MA20 (Figs 2 and 3). By contrast, for seeds exposed to *B. bassiana*, we detected *B. bassiana* growing endophytically within roots, stems, and leaves at both time points, and this was the case for both GHA and MA20 (Figs 2 and 3).

**Fig 4:**
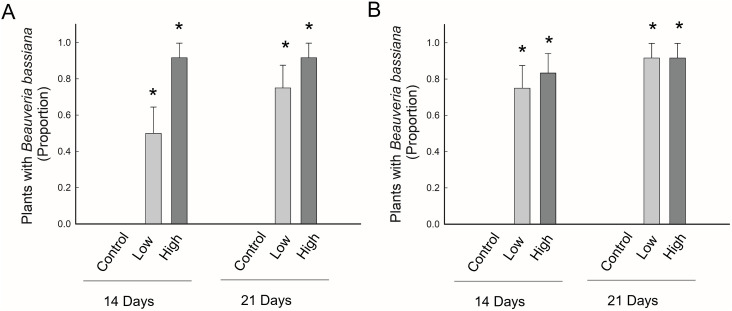
Endophytic occurrence in maize for two strains of *Beauveria bassiana:* **A) GHA and B) MA20.** Data are presented for two time point, 14 and 21 days after planting, and for three concentrations of *B. bassiana* conidia that were used to inoculate maize seeds: control = 0 conidia/seed, low = 3 × 10^8^ conidia/seed and high = 3 × 10^9^ conidia/ seed. Bar heights are the proportion of samples displaying endophytism, error bars are the standard error of the proportion. An asterisk indicates a significant difference from the control.

For experiments using GHA, *B. bassiana* was recovered significantly more from plants treated with conidia than the control plants, with the exception of leaf and stem tissues from the low conidia treatment (3 x 10^8^ conidia/seed), which were not significantly different from the control (Figs 2 and 4A). For seeds treated with conidia of GHA, the percentage of plants with endophytic presences of *B. bassiana* in roots was significantly greater for seeds treated with the high *vs* low concentration of conidia (Fig 2, Table 5). Additionally, the overall occurrence of endophytic *B. bassiana* was significantly greater for plants that received the higher treatment of conidia compared to plants that received the lower treatment (Fig 4A, Table 5). However, no significant effect of time or interaction between time and treatment was detected (Table 5). GHA was found growing endophytically in roots for 73% of the plants, which was significantly greater than the occurrence in stem tissue of 29% of plants (χ^2^ = 18.4; df = 1 P < 0.0001) and leaf tissue of 8% of plants (χ^2^ = 41.5, df = 1; P < 0.0001). Additionally, GHA occurred endophytically in stems significantly more than leaves (χ^2^ = 6.8; df = 1; P = 0.009) (Fig 2).

**Table 5:** Effect of treatment and time on the proportion of maize tissues colonized by *Beauveria bassiana* GHA.

Tissue	Effect	df	χ^2^	P
**Root**	Treatment^1^	1	3.96	0.047
	Time^2^	1	0.75	0.388
	Treatment × Time	1	0.08	0.783
**Stem**	Treatment	1	2.94	0.087
	Time	1	0.33	0.564
	Treatment × Time	1	0.33	0.564
**Leaf**	Treatment	1	0	1
	Time	1	0	1
	Treatment × Time	1	0	1
**Overall Presence**	Treatment	1	3.96	0.047
	Time	1	0.75	0.388
	Treatment × Time	1	0.75	0.388

^1^Treatment represents concentrations of *Beauveria bassiana* GHA applied to seeds: 3 × 10^8^ conidia/seed and 3 × 10^9^ conidia/seed.

^2^Time represents the two time points at which data were collected: 14 days after planting and 21 days after planting.

For experiments using MA20, *B. bassiana* was recovered significantly more from plants treated with conidia than the control plants, with the exception of *B. bassiana* recovered from the leaves at both concentrations at day 14, and from the low concentration at day 21 (Fig 3). Additionally, there was no significant difference in the percentage of plants that were positive for the presence of endophytic *B. bassiana* between the control and stems at the low concentration at day 14 (Fig 3). For seeds treated with MA20, there was no significant effect of concentration of conidia with which seeds were treated, time or interaction of these factors on the occurrence of endophytic *B. bassiana* for any plant tissue or for the overall presence in the plant (Fig 3 and 4B; Table 6). MA20 was found growing endophytically in roots for 81% of the plants, which was significantly greater than the occurrence in stem tissue of 40% of plants (χ^2^ = 17.4; df = 1 P < 0.0001) and leaf tissue of 19% of plants (χ^2^ = 37.5, df = 1; P < 0.0001). Additionally, MA20 occurred endophytically in stems significantly more than leaves (χ^2^ = 5.0; df = 1; P = 0.02). (Fig 3).

**Table 6:** Effect of treatment and time on the proportion of maize tissues colonized by *Beauveria bassiana* MA20.

Tissue	Effect	df	χ^2^	P
**Root**	Treatment^1^	1	0.08	0.773
	Time^2^	1	0.75	0.388
	Treatment × Time	1	0.75	0.388
**Stem**	Treatment	1	2.05	0.152
	Time	1	0.75	0.388
	Treatment × Time	1	0.75	0.388
**Leaf**	Treatment	1	0.02	0.883
	Time	1	0.61	0.433
	Treatment × Time	1	1.28	0.258
**Overall Presence**	Treatment	1	0.08	0.773
	Time	1	0.75	0.388
	Treatment × Time	1	0.08	0.773

^1^Treatment represents concentrations of *Beauveria bassiana* MA20 tested: 3 × 10^8^ conidia/seed 3 × 10^9^ conidia/seed.

^2^Time represents the two time points at which data were collected: 14 days after planting and 21 days after planting.

**Fig 5:**
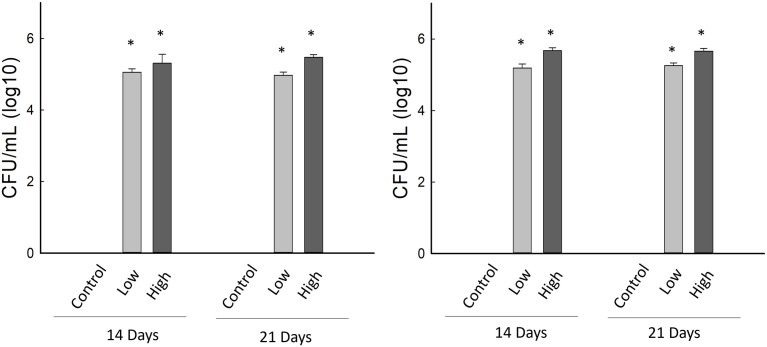
Colony forming units (CFU) per ml of vermiculite for two strains of *Beauveria bassiana:* **A) GHA and B)** **MA20**. Data are presented for two time point, 14 and 21 days after planting, and for three concentrations of *B. bassiana* conidia that were used to inoculate maize seeds: control = 0 conidia/seed, low = 3 × 10^8^ conidia/seed and high = 3 × 10^9^ conidia/ seed. Bar heights sample means, error bars are the standard error of the means. An asterisk indicates a significant difference from the control.

Colony-forming units for both GHA and MA20 were recovered from the vermiculite at both time points and for both concentrations tested (Fig 5, Table 7). For both GHA and MA20, the number of colony-forming units recovered from the vermiculite was significantly greater in plants that received conidia than those that did not, and this was the case for both concentrations at both time points (P = < 0.001 in all cases based on a Kruskal Wallis test) (Fig 5). Additionally, the concentration of colony-forming units increased significantly with the concentration of conidia applied to maize seeds for both GHA and MA20 (Fig 5, Table 7). However, the number of colony-forming units did not differ significantly over time, although there appeared to be a slight numeric increase between the first and second time points.

**Table 7:** Analysis of variance for colony forming units of *Beauveria bassiana* from GHA and MA20.

Isolate	Effect	df	F	P
**GHA**	Treatment^1^	1, 3	11.98	0.041
	Time^2^	1, 3	0.11	0.767
	Treatment*Time	1, 3	1.37	0.326
**MA20**	Treatment	1, 3	55.65	0.005
	Time	1, 3	0.04	0.848
	Treatment*Time	1, 3	0.33	0.608

^1^Treatment represents concentrations of *Beauveria bassiana* tested: 3 × 10^8^ conidia/seed and 3 × 10^9^ conidia/ seed.

^2^Time represents the two time points at which data were collected: 14 days after planting and 21 days after planting.

## Discussion

This study demonstrates the establishment of two strains of *B. bassiana*, GHA and MA20, as endophytes in maize when maize seeds were exposed to the fungal conidia, and the persistence of *B. bassiana* in the rhizosphere. *Beauveria bassiana*, for both strains, was recovered as an endophyte in roots, stems, and leaves of maize, and in association with the maize rhizosphere (Figs 2 and 5). Additionally, the presence of endophytic *B. bassiana* did not have a significant positive or negative effect on plant growth metrics (Tables 2 and 4). The presence of *B. bassiana* as an endophyte is aligned with previous studies conducted with *B. bassiana* in maize, however, this study also examined the pattern of colonization from the source of inoculation, the seed, to more distal portions of the plant tissue over time [6,7,28]. The persistence of *B. bassiana* in the rhizosphere and endophytically illustrates the potential compatibility of this entomopathogenic fungus with maize, and provides a more complete understanding of the pattern of fungal colonization and persistence [6,28].

For both strains, *B. bassiana* was found significantly more in the roots than stems or leaves, and more significantly more in the stems than leaves. While the endophytic presence of *B. bassiana* across these plant parts suggests that it does not remain localized to the exposure site, the process producing this pattern is unclear. For example, it may be the case that *B. bassiana* grows from the roots into more distal plant tissues, or alternatively, plant tissue may be colonized early in development with *B. bassiana* then persisting in these regions as the plant structures grow and expand. Past research has found that *B. bassiana* can establish as an endophyte through a number of inoculation methods, including seed soak, soil drench, root dip, or foliar application, and there is evidence that the inoculation method has an effect on the patter of colonization [15,35,36]. In general, the results of this study suggest that *B. bassiana* can be found as an endophyte throughout plant tissue irrespective of a single inoculation to the seed and that colonization throughout the plant changes over time.

Our study found endophytic persistence of *B. bassiana* for 21 days post inoculation and did not detect a significant decrease in endophytic prevalence of this fungus between 14 and 21 days after inoculation. In a study by Ramanujam et al., [37] persistence of *B. bassiana* decreased with the age of the of the plant when assessed for 90 days post treatment, suggesting that a reduction in the occurrence of endophytic *B. bassiana* likely would arise after the 21 day interval examined in this study. For annual crops such as maize, the short-term, stable endophytic persistence observed in this study may be beneficial in protection to seedlings from early season insect pests and plant pathogens. Variation in colonization and persistence may arise among strains of *B. bassiana*, or may be altered by host plant species, the presence of other fungal or bacterial endophytes, and nutrient availability [38–40]. However, for the *B. bassiana* strains examined in this study were found to have similar patterns of colonization and persistence.

The presence of *B. bassiana* did not significantly affect plant growth measurements in this study, and the lack of effect on plant growth differs from several studies that have found increased biomass production in various plants when exposed to entomopathogenic fungi including *B. bassiana* [19,24]. Tall and Meyling [17] found that *B. bassiana* significantly increased maize biomass only when nutrient levels were supplemented daily with fertilizer, but such effects were not seen when plants received only a single treatment of fertilizer. Because plants in this study were grown in vermiculite and only received fertilizer either once (plants measured 14 days after planting) or twice (plants measured 21 days after planting) during this study, this may have contributed to the lack of a significant effect on plant growth. As such, it is important to note that the lack of an effect of *B. bassiana* on growth of maize plants arose under the specific conditions of this study, which appear to include lower nutrient conditions than in other studies. Additionally, while the use of a sterile substrate in this study facilitated measurements of *B. bassiana* colonization and retention in the rhizosphere, it may have also affected interactions of *B. bassiana* with maize plants and the extent to which *B. bassiana* affected the growth of maize. Furthermore, the limited duration of this study, between 14 and 21 days, also may have limited our capacity to detect effects of *B. bassiana* on plant growth. However, it is also noteworthy that this study did not detect any adverse effects on plant growth metrics arising from the endophytic presence of *B. bassiana* (Tables 2, 3, 4).

Colony-forming units of *B. bassiana* were recovered from the growth medium (sterilized vermiculite) for plants treated with conidia, and this was the case through 3 wks after planting (Fig 4). No colony-forming units were recovered from vermiculite of control plants, and colony-forming units in the vermiculite also increased significantly with the increased concentrations of conidia applied to seeds and also appear to show a slight numeric increase over time (Fig 5). Root exudates have an essential role in the composition, establishment, and persistence of microbial communities in the rhizosphere [41].Findings by Mckinnon et al. [42] reported that *Beauveria* was more persistent in the rhizosphere of maize plants which were artificially wounded. Plant production of chemical signals in the rhizosphere, which may be induced in times of plant stress such as wounding caused by an herbivore, can shape the soil microbial community and mediate interactions with beneficial microbes such as entomopathogenic fungi [43,44].While it is not possible to determine the contribution of the rhizosphere to persistence of *B. bassiana* versus the level of persistence that may be possible in vermiculite alone, these data demonstrate the viable propagules of *B. bassiana* remained present in the rhizosphere for multiple weeks.

Even though *B. bassiana* was recovered from all maize tissues examined in this study, it is important to note that this work was conducted in sterile vermiculite, and the level of endophytism, and plant-fungal interactions in general, will be subject to effects of the naturally occurring microbial population in the field [2,15,29,41]. Many studies assessing the endophytism of *B. bassiana* in crops such as maize, tomato (*Solanum lycopersicum*), and beans (*Phaseolus vulgaris*) have been conducted in sterile substrates [22,23,45]. Studies that assessed endophytic colonization of plants by entomopathogenic fungi in non-sterile substrates found either a lack of establishment or a highly variable establishment [15,46]. A study by Rivas-Franco [47] showed that in the presence of a root feeding beetle larvae (*Costelytra giveni*) reduced colonization of maize roots by *B. bassiana* and *Metarhizium* spp., while the presence of a plant pathogen causing root rot (*Fusarium graminearum)* increased overall colonization. These studies demonstrate that the composition of the biotic community in the soil can influence plant interaction with entomopathogenic fungi in the rhizosphere. Plant-fungal interactions that begin in soil environments have additional complexities that arise from interactions with the surrounding microbial community, the soil microclimate, and other physical and chemical properties of a soil [1,2,41]. Some microbial communities may reduce the growth of entomopathogenic fungi through competitive exclusion and production of fungicidal compounds, while conversely, other communities may facilitate fungal growth and colonization of plant tissue [41]. Incorporating entomopathogenic fungi into seed coats, as done by Rivas-Franco et al. [47], or granules support their establishment by providing a substrate that may facilitate their growth. While using sterile substrates is beneficial in identifying entomopathogenic fungal strains capable of growing endophytically, it is essential to build on these studies with research that incorporates other field-relevant factors.

The presence of entomopathogenic fungi in the rhizosphere or as an endophyte may be beneficial to plants by providing protection from herbivorous pests and plant pathogens [25,27,48]. Maize plants colonized by *B. bassiana* have been shown to reduce herbivory by *Rachiplusia nu* (Lepidoptera: Noctuidae), as well as negatively affect the growth and reproduction of fall armyworm (*Spodoptera frugiperda*) [6,19]. In a study by Qin et al. [21], when *B. bassiana* was introduced as an endophyte to tobacco (*Nicotiana benthamiana*), plant growth increased, and there was increased resistance to an aphid pest (*Myzus pericae)* as well as to bacterial and fungal pathogens. By contrast, in bean (*Vinca faba*), soybean (*Glycine max*) and wheat (*Triticum aestivum*), researchers have found that the presence of insect pathogenic fungi, growing endophytically, resulted in an increased number of phloem-feeding pests [49–51]. While the mechanisms of protection are not well understood, one possibility is the induction of host-plant defenses, which has been reported in maize inoculated with *B. bassiana* [52]. A proteomic analysis in date palm (*Phoenix dactylifera* L.), inoculated with entomopathogenic fungi including *B. bassiana,* suggests that there was an upregulation in proteins that aid in plant defenses [53]. An important next step in the research reported here is to understand the effect of endophytic *B. bassiana* on interactions of maize with various agricultural pests and to explore the effect of *B. bassiana* strains on plant defenses when insects or plant pathogens are present.

The production of secondary metabolites by entomopathogenic fungi in the genera *Beauveria*, *Metarhizium*, and *Isaria*, can aid in the killing insect pests, however, it is less clear what role these insecticidal metabolites might play when the entomopathogenic fungi grow endophytically [54–58]. Some studies have shown that the endophytic presence of entomopathogenic fungi can make plants more resistant to plant pathogens and herbivores by causing changes in plant physiology and plant production of secondary metabolites [25,52,53]. A study Rasool et al.[51], which assessed both the endophytic colonization of wheat (*Triticum aestivum*) and bean (*Phaseolus vulgaris*) by *Metarhizium* and *Beauveria* species and the production of plant secondary metabolites, suggested that the effects on the insect pest was more likely due to the plant systemic response initiated by the entomopathogenic fungi rather than direct effects of the entomopathogenic fungi. However, secondary metabolites produced by entomopathogenic fungi have been found in plant tissue. A class of secondary metabolites produced by *Metarhizium* called destruxins, has previously been reported in cowpea (*Vigna unguiculata*), potato (*Solanum tubersum*), maize and bean (*P.vulgaris*), when artificially inoculated with *Metarhizium* [59–61]. Barelli et al. [61] found destruxin levels produced within maize and bean and levels of production varied by strain and host. While metabolites produced by entomopathogens may be produced within plant tissue, it remains an underrepresented area of research, and their relative contribution to mediating interactions between plants and herbivorous insects is not well understood. Additional studies assessing the production of secondary metabolites by entomopathogenic fungi within plant tissue, and the role of entomopathogenic fungi in upregulating plant secondary metabolites, could help to provide a more complete understanding of their interactions with plants.

Characterizing *B. bassiana* and other entomopathogenic fungi as endophytes of crops is an important first step in understand these multi-species interactions, and these characterizations can be carried out using selective media, molecular and histological approaches [62–64]. The use of selective media, as in this study, is a common method which provides a qualitative assessment of endophytic colonization of entomopathogenic fungi, which was the goal of our study. In a molecular approach by Liu et al. [65] B*. bassiana* and *M. anisopliae*, both of which increased various plant growth measurements, were confirmed to grow endophytically in maize using both selective media and detection of fungal DNA based on polymerase chain reaction (PCR), with the PCR method allowing for greater sensitivity in detection than selective media. While both selective media and PCR-based approaches can confirm endophytic colonization, histological approaches using light and scanning electron microscopy can show how entomopathogenic fungi are able to penetrate and colonize plant tissue. For example, Wanger and Lewis [63] used light and scanning electron microscopy to demonstrate how *B. bassiana* penetrates the epidermis of maize leaves to colonize endophytically. Conversely, a similar approach by Koch et al.[64] assessed four entomopathogenic fungi, *B. bassiana*, *M. anisopliae*, *I. fumosorosea* and *Trichoderma harzianum,* in four host plants including maize, but did not find successful colonization. While it is unclear why colonization was unsuccessful, a study by Ullrich et al.[66] found local colonization B*. bassiana*, *M. anisopliae*, *I. fumosorosea* fungi in *V. faba* and *Cucumis sativus* plants but suggested that variable colonization could be due to plant stress responses to hyphal penetration. In a combination of molecular and histological approaches, Landa et al. [67] developed a qPCR method of detecting *B. bassiana* and labeled the fungi with green fluorescent proteins to assess the distribution and concentration of fungal endophytes over time, providing a quantitative assessment. While the methodology must be specific to the research question, each of these approaches contributes to the comprehensive understanding of entomopathogenic fungi as endophytes.

This study illustrates that the entomopathogenic fungus *B. bassiana* can colonize maize plants and survive outside of an insect host. Additionally, this study adds to the understanding of the interactions between entomopathogenic fungi and maize, particularly in terms of persistence in the rhizosphere and the pattern of endophytic colonization. Future research exploring effects of endophytic entomopathogenic fungi, and fungal persistence in the rhizosphere, on plant growth and interactions with agricultural pests may help to increase agricultural productivity and sustainability [1,25,68].
